# Subpial hemorrhages in neonates: imaging features, clinical factors and outcomes

**DOI:** 10.1038/s41598-023-30332-5

**Published:** 2023-02-28

**Authors:** Xiamei Zhuang, Ke Jin, Junwei Li, Yan Yin, Siping He

**Affiliations:** Department of Radiology, Hunan Children’s Hospital, 86 Ziyuan Road, Yuhua District, Changsha, China

**Keywords:** Diseases, Neurology

## Abstract

Neonatal subpial hemorrhage is a poorly understood type of intracranial hemorrhage. Herein, we reported on 34 neonates with subpial hemorrhages, focusing on the imaging features, clinical factors, and outcomes of this type of intracranial hemorrhage. This retrospective case series enrolled 34 neonates with subpial hemorrhages. We analyzed their magnetic resonance (MR) images, clinical manifestations, and prognoses. We categorized, for the first time, the MR images of patients with subpial hemorrhages into three imaging patterns; moreover, on the basis of a yin-yang sign, we added a sandwich sign, attaining an MR image feature that was easier to understand. MR Patterns A and B both have good prognoses, and most patients had normal clinical outcomes. Subpial hemorrhage in neonates may be diagnosed via imaging patterns. Recognizing this pattern of hemorrhage may help gain a better understanding of the associated risk factors.

## Introduction

Subpial hemorrhagic stroke is a form of intracranial hemorrhage primarily in neonates that has been largely ignored until recently^1–3^. Subpial hemorrhage was first described in infant autopsies^4^, followed by autopsy reports 1928 and 1934^5,6^. Brain magnetic resonance (MR) imaging (MRI) of neonates has facilitated the early detection of subpial hemorrhages. Huang (2004) confirmed seven cases of subpial and superficial parenchymal hemorrhage via MRIs^2^. Subsequently, few cases of this condition were reported. However, the inducement, pathology, and prognosis of subpial hemorrhage are unknown, and relatively few imaging studies have been conducted. With the increasing popularity of MRI in neonates, those previously treated for subarachnoid hemorrhage and parenchymal hemorrhage did not have this type of hemorrhage. Therefore, in this study, we retrospectively analyzed the clinical and imaging data of 34 newborns with subpial hemorrhage to improve our understanding of this condition.

## Methods

This single center study was conducted according to the principles expressed in the Declaration of Helsinki. The Medical Ethics Committee of the Hunan Children’s hospital of the University of South China approved this retrospective study and waived the requirement for informed consent.

Electronic medical records of all neonates with an MR imaging diagnosis of subpial hemorrhage were reviewed. Laboratory values, clinical course, imaging indications, and prognostic data were extracted from the medical records. Extracted data included gender, gestational age, birth weight, mode of delivery, Apgar score, initial laboratory tests, clinical presentation on admission, age of onset of symptoms, neurological diagnosis, and documentation of developmental delay after discharge.

### Patients

A pediatric neuroradiologist retrospectively identified the patients using our hospital’s computer system. We patients with subpial hemorrhage following a retrospective review of clinical images obtained from a clinical picture archiving and communication system (PACS), defined as (1) hemorrhage closely opposing the underlying sulci; (2) pooling of blood products relatively localized rather than spreading along the convexity^7^; (3) axial T1WI, FSE T2WI, fluid-attenuated inversion recovery (FLAIR), diffusion-weighted imaging (DWI), susceptibility-weighted imaging (SWI), and sagittal T1WI sequence examinations. An electronic medical chart was used to collect data pertaining to the patients’ demographics, clinical course, and laboratory findings.

### MRI protocol

In all cases, the first cranial MRI was performed within 30 days of birth. Nineteen patients underwent follow-up brain MRIs from 10 days to 3 years of age. MRI of the brains was performed at Hunan Children's Hospital between January 2016 and December 2021 for medical reasons. Scanners ranged from 1.5 to 3.0 T field strength. The MRI and scanning parameters varied; however, uniform anatomic sequences were used throughout the study, including axial T1WI, FSE T2WI, FLAIR, DWI, SWI, and sagittal T1WI. Time-of-flight MR angiography (MRA) and 2D phase non-contrast MR venography (MRV) were performed in some cases.

### Image analysis

Two pediatric radiologists, blinded to the clinical presentation and outcomes, conducted the analyses. If their results differed, they arrived at a consensus via discussion. In the retrospective PACS analysis, subpial hemorrhage and underlying brain parenchyma were analyzed for the presence, location, and intensity of the signal. All available MRA and MRV images were reviewed to assess the diagnostic performance of the MRI modality. Follow-up MRIs were conducted to evaluate the natural progression of the disease.

## Results

### Patients

Thirty-four patients were enrolled at our hospital (Department of Neonatology) between January 2016 and January 2022. The patient demographics are summarized in Table 1. Twenty-one patients were male and 13 were female (ratio = 1.62). The median age at disease onset was 11 days (range, 5–28 days). Most (85.3%) were born at term gestation ($\ge 37 \mathrm{weeks}$), four were moderate-to-late preterm (32–37 weeks), and one was very preterm ($\le$ 32 weeks). The majority (85.3%) of deliveries were vaginal, and most were of birth weights > 2.5 kg.

**Table 1:** Patient demographics, clinical presentation, and outcomes.

Clinical Parameters	Subgroups	Values
Gestational age at birth(weeks)	Term (< 37)	29/34 (85.3%)
	Moderate-to-late preterm (32–37)	4/34 (11.8%)
	Very preterm (> 32)	1/34 (2.9%)
Sex	Male	21/34 (61.8%)
	Female	13/34 (38.2%)
Mode of delivery	Vaginal	29/34 (85.3%)
	Cesarean delivery	5/34 (14.7%)
Birth weight	< 2.5 kg	7/34 (20.1%)
	> 2.5 kg	27/34 (79.4%)
Imaging indication	Asphyxia	7/34 (20.1%)
	Jaundice	14/34 (41.2%)
	Cyanosis	5/34 (14.7%)
	Fever	3/34 (8.8%)
	Seizure	1/34 (2.9%)
	Dyspnea	4/34 (11.8%)
APGAR score at 1 min		7.61 (range,1–10)
APGAR score at 5 min		9 (range, 3–10)
APGAR score at 10 min		9.76 (range, 4–10)
APGAR score is unknown		8/34
Coagulation abnormalities (first 7 postnatal days)	AT3 (80–120%)	25/32 56.16% (range, 23–79%)
	FDP (0–5 μg/ml))	16/32 16.65 μg/ml (range, 5.92–40.69 μg/ml)
	D-D (0–0.5 μg/ml))	12/32 6.41 μg/ml (range, 0.76–25.56 μg/ml)
	PT (10–14 s)	10/32 20.13 s (range, 14.6–31.0 s)
	APTT (28–48 s)	8/32 55.96 s (range, 48.8–68.8 s)
	FIB (170–450 mg/dL)	8/32 372.75 mg/dL (range, 41–683 mg/dL)
	TT (14-20 s)	5/32 28.28 s (range,21.9–35.2 s)
	INR (0.8–1.5)	2/32 2.85 (range, 2.72–2.97)
Age at onset of symptoms		11d (range, 5-28d)
Age at most recent follow-up in months, median		29 (3–62)
outcome	Died	1/29
Abnormality at most recent follow-up	Dyskinesia	7/28 (25.0%)
	Developmental retardation	5/28 (17.9%)
No neurologic deficits		16/28 (57.1%)

*AT3* antithrombin, *APTT* activated partial thromboplastin time, *D-D* D-Dimer, *PT* prothrombin time, *INR* international normalized ratio, *FDP* fibrinogen degradation products, *FIB* fibrinogen, *TT* thrombin time.

### Imaging indication, comorbid conditions and outcomes

Imaging indications are listed in Table 1. The most common indications were jaundice (41.2%) and asphyxia (20.1%). Comorbid conditions included hyperbilirubinemia in 14 patients, neonatal pneumonia in 8, hypoxic-ischemic encephalopathy in 5, neonatal bacterial meningitis in 3, congenital heart disease in 3, and cardiorespiratory failure in 1. Neonatal scalp hematomas were common (24/34, 70.6%). Thirty-two patients were examined for coagulation function within 7 days, and acute coagulation abnormalities were common, with the most frequent being elevated antithrombin (25/32, 78.1%) and elevated fibrin degradation products (16/32, 50.0%). Only one patient was diagnosed with a hypercoagulable disorder, while four patients were diagnosed with disseminated intravascular coagulation. We obtained APGAR scores for 26 neonates (21 term, 5 preterm). No child had any serious congenital malformation.

Perinatal history was generally unremarkable. The following pregnancy complications were observed in our cohort: chorioamnionitis (n = 3), oligohydramnios (n = 1), preeclampsia (n = 1), and gestational diabetes mellitus (n = 1). Two of these were twin pregnancies.

Follow-up information was available for 26 patients (range, 3–62 months). One patient died after discharge: a term female infant with massive hemorrhages in the left temporal and occipital lobes. Twelve patients yielded abnormal neurological examination findings at discharge (42.9%), 25.0% of whom had dyskinesia and 17.9% had developmental retardation. The 16 remaining patients survived and showed good outcomes at a median of 27 months; none had residual seizures, neurologic deficits, or developmental issues. All patients with dyskinesia had at least one other type of concurrent intracranial hemorrhage. Of the three neonates with isolated subpial hemorrhage, one had developmental retardation (only isolated language delay), and the remaining two showed good outcomes. Among the remaining four follow-up patients with developmental disorders, one had intracranial hemorrhage, one had arterial ischemic stroke, and the other two had an underlying cerebral infarct.

### Treatment

For subpial hemorrhage, 29 patients received conservative treatment only, such as intravenous injection of vitamin K1 or etamsylate. Five cases were incorrectly identified as having subdural hemorrhage, and the bleeding site was surgically drained on the basis of conservative treatment.

### MRI findings

The results of the initial MRI scans are summarized in Table 2. Most lesions were single and unilateral (85.3%), and five were multiple. Multiple lesions can be bilateral or unilateral. In most cases, lesions were found in the temporal lobe (52.9%), followed by the occipital lobe (29.4%), frontal lobe (20.1%), and parietal lobe (20.1%); some larger lesions can be distributed across multiple lobes. No lesions were observed in the posterior fossa. On MRI, 85.3% patients had subarachnoid hemorrhage, 26.5% patients had subdural hemorrhage, and 64.7% patients had parenchymal hemorrhage. No midline shift or uncal herniation mass effect was observed in any of the cases, but all showed some degree of regional mass effect with effacement of the adjacent sulci.

**Table 2:** MR imaging data at presentation.

	No	Percentage
Single	29	85.3
Multiple	5	14.7
Location		
Temporal	18	52.9
Frontal	7	20.1
Parietal	7	20.1
Occipital	10	29.4
Subarachnoid hemorrhage	29	85.3
Subdural hemorrhage	9	26.5
Parenchymal hemorrhage	22	64.7
Intraventricular hemorrhage	8	23.5
Subjacent cortical infarction	28	82.4

Eighteen of the 34 patients underwent MRA, and five had MRV completed within 5 days of their initial MR scan. On one MRA, there was evidence of thrombosis in the left middle cerebral artery, but none of the MRV showed evidence of thrombosis in either the dural venous sinus or cerebral veins.

Based on the T2-weighted imaging and DWI appearance of the subpial hemorrhages with or without subjacent cortical infarction or parenchymal hemorrhage, we identified three different patterns of findings (Table 3).

(A) Subpial hemorrhage only
(B) Subpial hemorrhage with subjacent cortical infarction but without parenchymal hemorrhage
(C) Subpial hemorrhage with subjacent cortical infarction and parenchymal hemorrhage

**Table 3:** MR imaging features of subpial hemorrhage in neonates.

	Fluid–fluid level		Restricted diffusion			Yin-yang sign	sandwich sign
	Subpial Hemorrhage	Parenchymal Hemorrhage	Subpial Hemorrhage	Cortex	WM		
Pattern A (6/34)	2	0	2	0		4	2
Pattern B (5/34)	3	0	0	5	0	5	0
Pattern C (23/34)	10	1	2	23	16	3	20

### Pattern A

**Figure 1:**
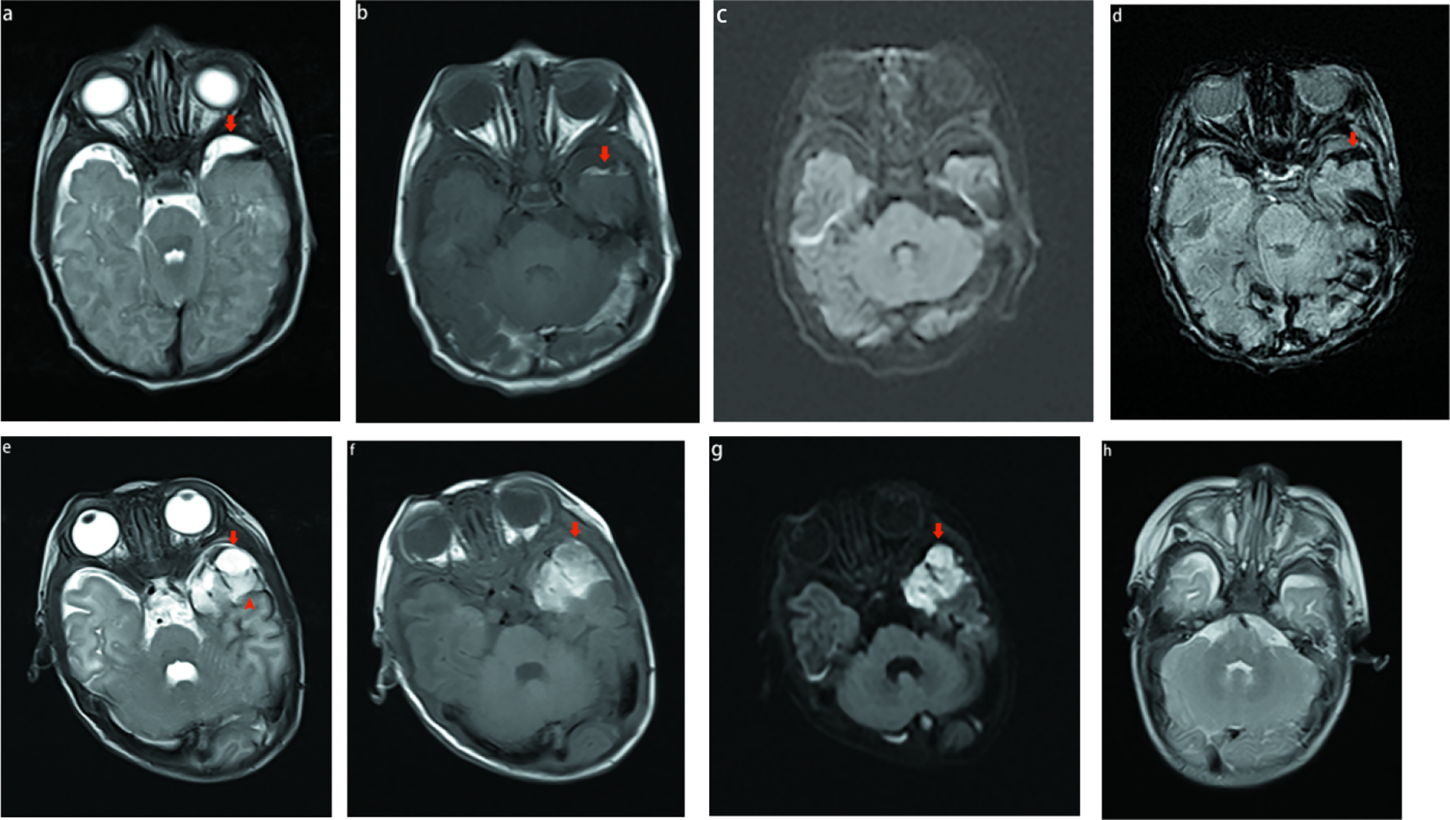
MR images of 2 full-term neonates with pattern A. In the first patient (**a**–**d**), the subpial hemorrhage shows hepointense on T2WI (**a**), hyperintense on T1WI (**b**), no restricted diffusion (**c**), and hypointense signal on SWI (**d**). The combination of a dark or bright subpial hemorrhage collection and the normal underlying cerebral cortex forms a yin-yang symbol (**a**–**b**). In the second patient (**e**–**g**), the subpial hemorrhage shows hyperintense on T1WI and T2WI, with restricted diffusion (**g**). On T2WI (**e**), we found a thin layer of additional hypointense fluid in the deep aspect between hemorrhage and the cortex (arrowhead), the presence of a bright subpial bleed, a dark, thin layer of fluid and an underlying normal cortex-created sandwich sign. The first patient received MRI examination after 3 months; The subpial hemorrhage was completely absorbed and the cerebral sulcus fissure widened (**h**).

Six of the 34 patients had the first imaging pattern. Owing to different bleeding times, the signal performance of each sequence was different. Subpial meningeal hemorrhage may be either hyperintense or hypointense on T1 weighted and T2 weighted images. Only 2/6 showed restricted diffusion, and 2/6 subpial hemorrhages showed fluid–fluid levels. The different signals between the subpial hemorrhage and the subjacent cortical areas created a distinct MRI characteristic, characterized by the presence of dark or bright subpial bleed and normal cortical signals. Similar to previous studies, the mass effect and unique imaging feature of cortical inward depression (cortical buckling) created a yin-yang symbol^8^. Figure 1, particularly in T2WI and DWI. In this pattern, four patients had the yin-yang sign. Deep hemorrhage and cortex may be found with additional hypointense fluid on T2WI and DWI. The consistent presence of a bright, hyperintense subpial bleed, dark thin fluid layer, and an underlying normal cortex created another distinct MRI characteristic, resembling a sandwich sign (Fig. 1).

### Pattern B

**Figure 2:**
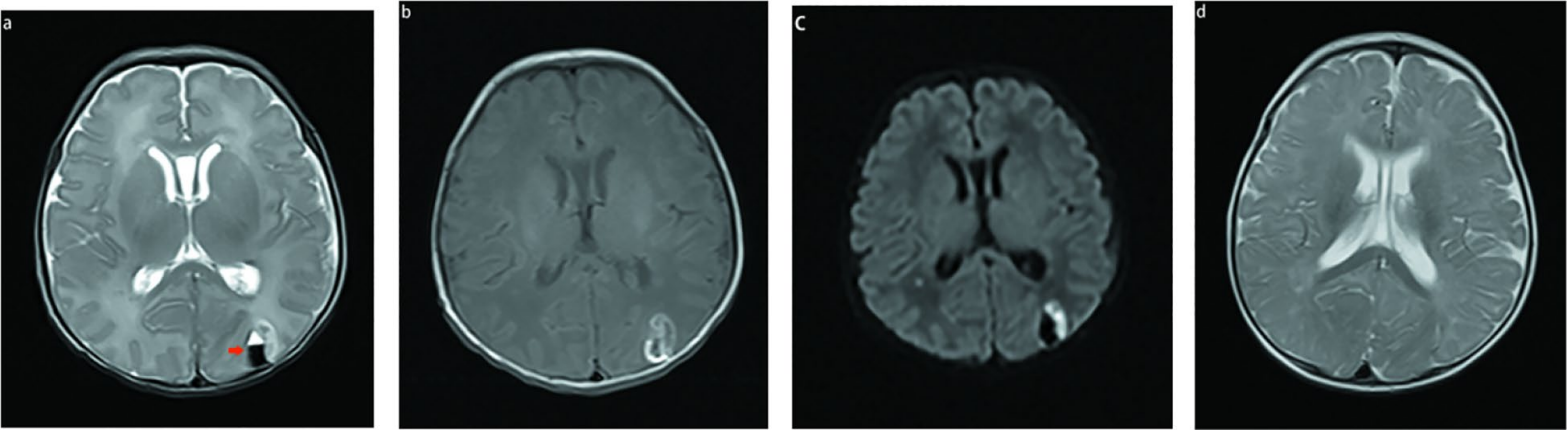
MR image of a full-term neonates with pattern B. The first MRI was performed at 5 days after birth (**a**–**c**). The subpial hemorrhage shows hypointense on T2WI and adjacent cortical infarction shows hyperintense (**a**), we found fluid–fluid level on subpial hemorrhage(arrow). Mixed signal on T1WI (**b**). There was no restricted diffusion in subpial bleed and hyperintense on cortical infarction (**c**). This combination of a dark subpial hemorrhage collection and the bright underlying cerebral cortex forms yin yang sign (**a** and **c**). Reexamination after 8 months showed that the subpleural hemorrhage had been basically absorbed, and there was no cortical infarction signal (**d**).

The second pattern was observed in five patients. In this pattern, the signal change in the adjacent cortex was crucial. It could include the cortex as well as the subcortices. In these cases, the entire cortex underneath the subpial hemorrhage demonstrated restricted diffusion (Fig. 2). Five cases had the yin-yang sign and none had the sandwich sign. On three of the five MRI scans, the subpial hemorrhage showed a fluid–fluid level.

### Pattern C

**Figure 3:**
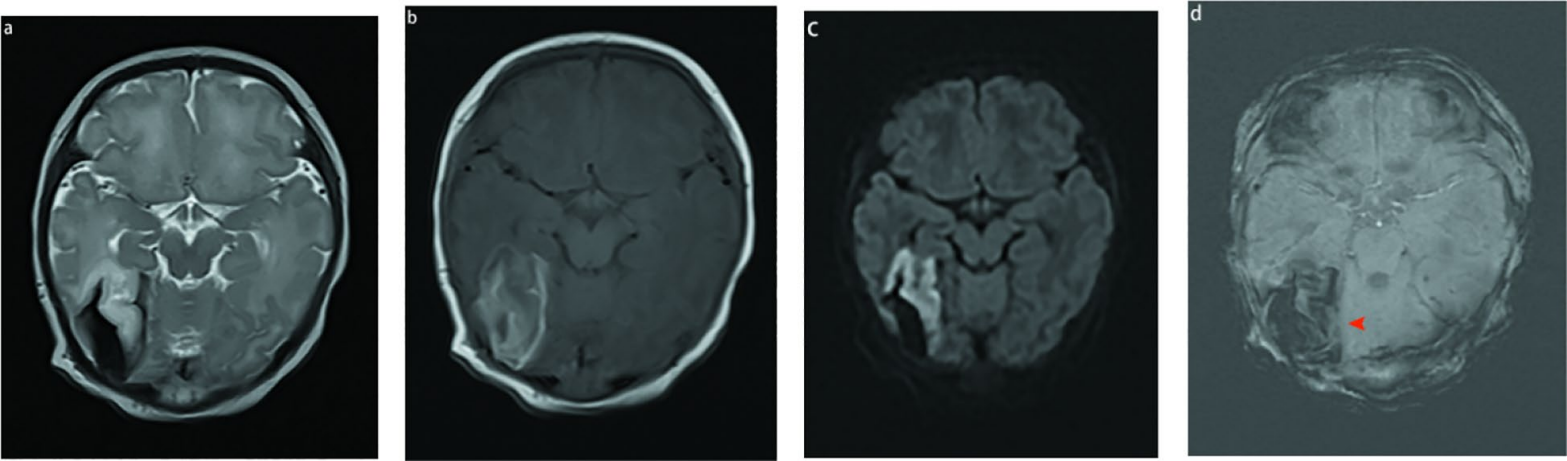
MR image of a full-term neonates with pattern C. The subpial hemorrhage shows hypointense on T2WI and hyperintense on T1WI. Hemorrhage is seen in the underlying cerebral cortex and white matter, resulting in a hyperintense on T1WI and T2WI (a-b). There was no restricted diffusion in the subpial bleed and hyperintense on underlying cerebral cortex and white matter (c). SWI shows magnetic susceptibility artifact blurs, making it diffcult to separate the subpial hemorrhage from underlying the cortical ribbon ( d, arrowhead). This combination of a dark subpial hemorrhage collection and the bright underlying cerebral cortex and white matter forms a yin-yang sign (a and c).

**Figure 4:**
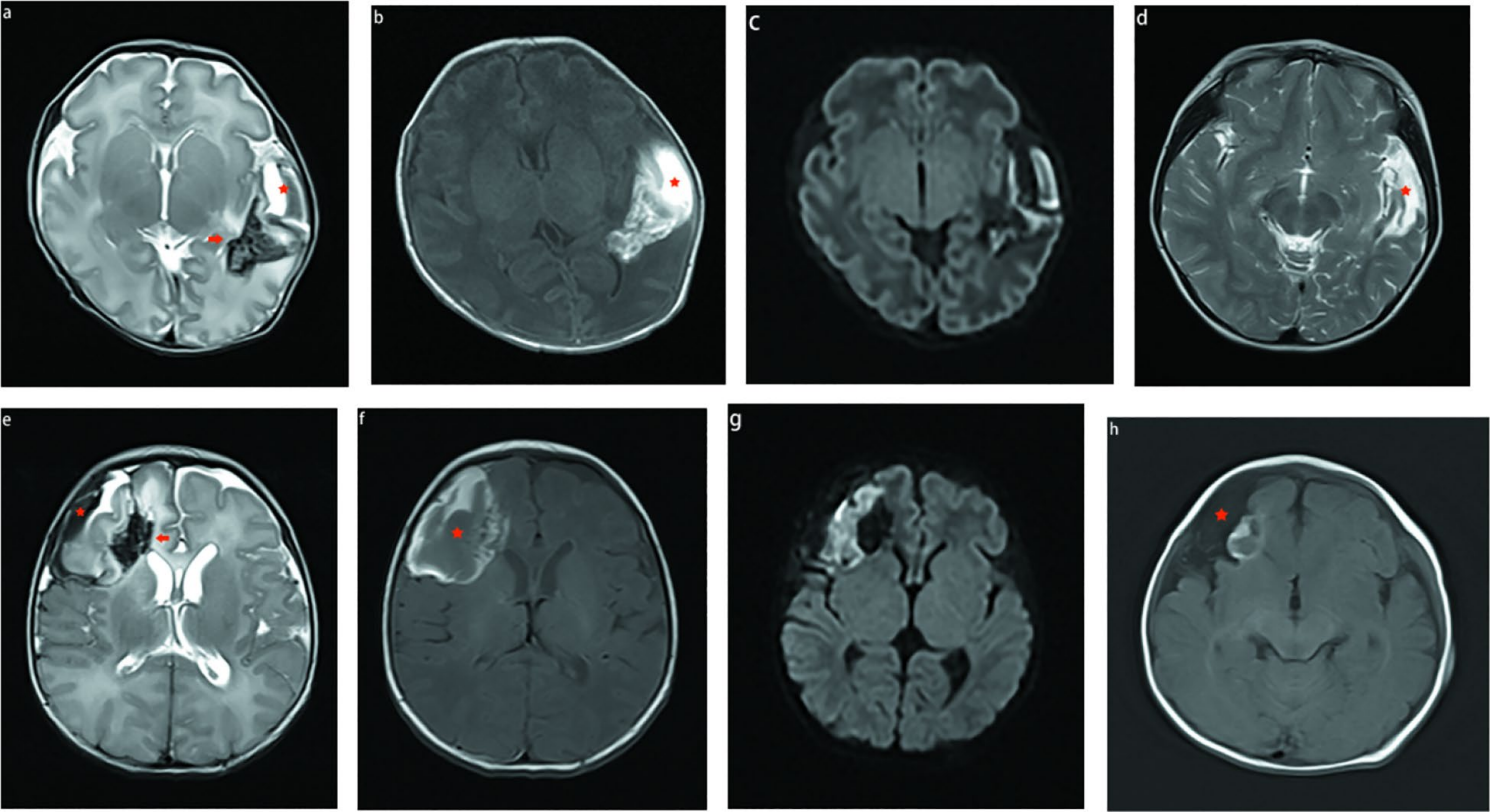
MR images of 2 neonates. The first patient (**a**–**d**) is a preterm neonate, and the second patient (**e**–**h**) is a term neonate. The first MRI shows (**a**–**c** and **e**–**g**) a subpial bleed (star), underlying the cerebral cortex and white matter with fan-shaped hemorrhage (arrow) created a sandwich sign. We found the medullary veins were notably enlarged (arrow in **a** and **e**). Reexamination after 3 years (**d**) and 5 months (**h**) showed that the subpleural hemorrhage and cerebral parenchymal hemorrhage had been absorbed and eventually encephalomalacia and gliosis. Subpial cystic cavities were formed (red star in d and h).

The third pattern was observed in 23 patients. On T1WI, T2WI, and DWI, the signals of the subpial hemorrhage and subjacent parenchymal hemorrhage were complex and variable because of the different bleeding times. The subpial collection demonstrated both hyperintensity and hypointensity on T1WI and T2WI, but only two patients showed restricted diffusion. Ten of the 23 MRI scans showed fluid–fluid levels. The main manifestations of parenchymal hemorrhage on T1WI, T2WI, and DWI were heterogeneous signals, and the involved cortex was predominantly hyperintense on T2WI and restricted diffusion imaging (Figs. 3 and 4). In this pattern, the signal of the subpial hemorrhage was completely different from that of the adjacent parenchymal hemorrhage on T2WI, so the subpial hemorrhage, underlying cerebral cortex, and adjacent white matter hemorrhage created the yin-yang sign or sandwich sign. If the involved cortex and white matter signal changes were consistent without a dark, thin fluid layer, they were visible as a yin-yang sign (n = 3) (Fig. 3); otherwise, they presented as a sandwich sign (n = 20) (Fig. 4).

### Other findings

T1WI, T2WI, and FLAIR are routine neonatal brain MRI protocols, and DWI and SWI have also become part of the repertoire. Subjacent cortical infarction was demonstrated predominantly by hyperintensity on T1WI, and when subpial hemorrhage also demonstrated hyperintensity on T1WI, the interface between the cortex and adjacent subpial hemorrhage could have been misinterpreted as subdural hemorrhage or parenchymal hemorrhage. Therefore, we could not identify the margins of the cortical buckling (Figs. 3 and 4). Compared to other sequences, SWI better visualizes hemorrhages; however, this technique can also yield misleading results because the magnetic susceptibility artifact blurs the interface between the adjacent infarct and subpial hemorrhage and may suggest an intraparenchymal hemorrhage in the peripheral tissue (Fig. 3). We could clearly visualize the interface between the cortex and adjacent subpial hemorrhage using T2WI and DWI.

In Pattern C, 9 cases of subpial hemorrhage were accompanied by superficial medullary vein engorgement and 12 were accompanied by deep medullary vein engorgement. Two cases of subpial hemorrhage were accompanied by superficial medullary vein engorgement and deep medullary vein engorgement.

### Follow-up MRI

Follow-up MRI was performed in 19 of the 34 patients, including one, two, three, and five follow-up scans in nine, three, one, and one patients, respectively. The last appearance of subpial hemorrhage on the follow-up MRI scans are summarized in Table 4.

**Table 4:** The last follow-up MR imaging of subpial hemorrhage in neonates.

	Time interval between initial and last follow-up MR imaging	No. of scans	The last follow-up MR imaging (appearances compared with initial MRI)			
			subpial hemorrhage	underlying parenchymal	widening of cerebral sulcuses and fissures	subpial cystic cavities
Pattern A (2)	1 mon	1	Evolved into fluid collection and absorb with less mass effect	Normal	Yes	No
	3 mon	1	Complete absorb with no mass effect	Normal	No	No
Pattern B (3)	1 mon	1	Evolved into fluid collection and absorb with less mass effect	Normal	Yes	No
	2 mon.–7mon	2	Complete absorb with no mass effect	Normal	No	No
Pattern C (14)	8 day–12 day	2	Similar size and shape	Similar size and shape	Yes	No
	20 day–2 mon	4	Evolved into fluid collection and absorb with less mass effect	Decreased size	Yes	2
	3 mon.–3 years	8	Complete absorb with less or no mass effect	Encephalomalacia and gliosis	Yes	4

## Discussion

Although subpial hemorrhage is pathologically distinct from subarachnoid and subdural hemorrhages, this type of intracranial hemorrhage is poorly recognized^9^. We reported on 34 neonates with subpial hemorrhages, focusing on the imaging features, clinical factors, and outcomes of this type of intracranial hemorrhage.

We analyzed the features of neonates' subpial hemorrhages on MRI in our cohort; subpial hemorrhages often exhibited concurrent subarachnoid (85.3%) and parenchymal hemorrhages (64.7%), which was similar to that reported by Assis et al.^8^ Of 34 total hemorrhages, 18 (52.9%) were in the temporal lobe. Compared with that reported in previous studies, the incidence of subpial hemorrhage located in the temporal region is slightly lower (69% and 82%)^8,10^. A previous study proposed that subpial hemorrhage underlies an impermeable pia mater, preventing prompt resorption of the blood and causes a degree of cortical damage, analogous to what occurs in compartment syndromes^3^. The local space-occupying effect can cause secondary compression of subpial vessels. Specifically, subpial veins are more susceptible to this local pressure. Within the subpial space, arteries are coated by a largely continuous single layer of pial leptomeningeal cells. In contrast, the veins in the subpial space are not encased in a leptomeningeal sheath^11^. When the pressure increases due to hemorrhage at the subpial space, the subpial veins are more susceptible to this local pressure. Therefore, subpial hemorrhage causes local venous congestion and hypertension due to blockage of cortical venous congestion, and hypertension due to the blockage of cortical venous outflow. The result is focal cortical or subcortical infarction characteristic of subpial hemorrhage^12^, or even superficial medullary venous congestion infarction. Subpial hemorrhage can also be accompanied by deep medullary venous infarction. The deep medullary veins mainly drain the superior choroidal venous blood, thalamostriate venous blood in the subependymal area, and caudate venous blood. It may also drain part of the intracortical venous blood through transcerebral veins. Therefore, abnormalities in deep medullary veins may play a key role in the pathogenesis of neonatal subpial hemorrhage^7,8,13,14^. In this cohort study, 21 patients with subpial hemorrhage had accompanying deep medullary vein engorgement and/or superficial medullary veins, indicating the change in venous pressure as a potential pathogenesis of subpial hemorrhage.

To our knowledge, this study is the first to identify three different patterns of MRI findings (Table 3). Pattern A was defined as subpial hemorrhage only, comprising typically ellipsoid, semielliptic, or spherical blood collections along the margins of the cerebral parenchyma, with the long axis tangential to the brain. The pia matter comprises a single thin layer of cells surrounding the cortex, reflecting itself on the surface of both the subarachnoid vessels and trabeculae. In this configuration, the cerebrospinal fluid-containing subarachnoid space is mechanically separated from the collagen-containing subpial space^12^. Hemorrhage usually occurs at the gyral and sulcal edges, and is sufficiently contained to produce a local mass effect (Fig. 1). Pattern B was defined as a subpial hemorrhage with adjacent cortical infarction only. In this case, cortical infarction may be due to the obstruction of superficial cortical venous reflux caused by a mass effect from the subpleural hemorrhage (Fig. 2). Pattern C was defined as a subpial hemorrhage with adjacent cortical infarction and subjacent parenchymal hemorrhage. Pattern C was the most common in our cohort. In this pattern, most medullary veins were enlarged, indicating congestion and thrombosis (Fig. 4). In our cases, no MRVs demonstrate dural venous sinus thrombosis, but the neonatal medullary venous congestion or compression is shown well in our cases. It may be due to the fact that MRV can show venous thrombosis in small veins larger than 1 mm^15^.

Through the expression of different signals between the subpial hemorrhage and adjacent cortical, subcortical, and white matter tissues, we identified distinct imaging patterns that resembled the yin-yang and sandwich signs. This characteristic image representation was further improved. In our cohort, 12 patients exhibited the yin-yang sign and 22 exhibited the sandwich sign. It may be easier for radiologists and clinicians to recognize these biomarkers during imaging. Imaging changes associated with bleeding over time should also be considered. For example, the appearance of the yin-yang sign and sandwich sign was largely due to the different timing of hemorrhaging under the subpial and adjacent cerebral parenchyma. However, the time of subpial hemorrhage demonstrably differs from that of cerebral parenchymal hemorrhage.

A series of MRIs was used to study disease progression (Table 4). On the follow-up MRI, despite the fact that all 19 cases showed that the subpial hemorrhage could be completely absorbed, subpial cystic cavities were formed in six cases. Widening of the cerebral sulcal fissures and underlying infarct disappeared after 7 months without parenchymal hemorrhage. Cerebral parenchymal hemorrhages slowly absorb over time and eventually develop into encephalomalacia and gliosis. The subpial hemorrhages did not progress with time, but the parenchymal hemorrhages did. This observation may help guide follow-up imaging.

In our cohort, 85.3% of the neonates were born by vaginal delivery, 79.4% weighed more than 2.5 kg, and 70.6% had neonatal scalp hematoma. Previous studies have shown that most patients are born by vaginal birth^2,8,16^; therefore, trauma may be related to subpial hemorrhage (traumatic pial-glial disruption, erythrocyte diapedesis). However, Cole confirmed that the neonatal hemorrhagic stroke is associated with a lack of trauma^17^. Other proposed risk factors associated with subpial hemorrhages include neonatal asphyxia, fetal head molding, clotting disorders, venous sinus compression, variations in intracranial pressure, and incomplete regression of the primary vascular network^18^. The most common imaging indication was jaundice (41.2%) and asphyxia (20.1%), but the infants in our cohort had variable clinical presentations including hyperbilirubinemia, neonatal pneumonia, hypoxic-ischemic encephalopathy, and neonatal bacterial meningitis. Few of our patients had been diagnosed with hypercoagulable disorders or disseminated intravascular coagulation. Whether the underlying diseases in our cohort were directly related to subpial hemorrhages or the result of the associated neurological pathologies was unclear.

Only one patient died because of massive parenchymal hemorrhage. At a median age of 27 months, more than half (61.5%) of the patients had good outcomes. Similar to the proportions reported in previous studies, 26.9% of patients had dyskinesia and 19.2% had developmental retardation^2,8^. The data from the present study is insufficient to determine whether surgical drainage for subpial hemorrhage is beneficial for long-term neurological evolution. In our group, the prognoses of most isolated subpial hemorrhages (patterns A and B) were good but became poor when combined with cerebral hemorrhage (Pattern C). The prognosis of partial subdural hemorrhage with subcortical infarction (Pattern B) was poor. The pia mater and glia limitans are normally closely apposed. The integrity of the pial-glial interface is an essential part of normal cortical development during the prenatal, neonatal, and postnatal periods. Therefore, subpial hemorrhages in these related structures can lead to serious developmental consequences. These cytoarchitectural changes, caused by subpial abnormalities, can lead to cortical dysfunction and clinical consequences. Cerebral parenchymal hemorrhages eventually lead to encephalomalacia and gliosis, and most patients experience dyskinesia. Therefore, neonatal subpial hemorrhage should be considered whether adjacent cortical infarction or parenchymal hemorrhage is present. Consequently, identifying the patterns of MRI findings in neonatal subpial hemorrhage is crucial.

This study had several limitations: (1) The results may be confounded by complications and other concomitant neurological injuries. (2) Our study lacked a standardized assessment delay and the sample size was too small to truly understand the impact of isolated subpial hemorrhage. (3) Asymptomatic cases without neuroimaging indications will be missed, which will bias our cohort toward more severe clinical presentations and prognosis. (4) Lack of a control group to determine the true impact of subpial hemorrhage. (5) retrospective study design, and (6) limited follow-up information. Since we collected clinical information on all neonates with subpial hemorrhage, our study should be considered a preliminary assessment and further evaluation is definitely needed. We are aware of the limitations of our study. Given the clinical heterogeneity of our sample, a larger cohort is needed to control for confounding factors and to truly understand the contribution of subpial hemorrhage to neurological deficits. Prognosis was assessed by a professionally trained clinician based on the Psychological Developmental Scale of Childhood Neurodevelopment and Gesell intellectual check based on follow-up data at a fixed time after hospital discharge. Complete long-term neurological outcomes can be collected. Future studies should be compared with controls, and all newborns routinely undergo study MRI as standard time span with special attention to identifying subpial hemorrhage, in the hope that it may help to increase awareness of this condition and improve its recognition in clinical practice. This has the potential to overcome these limitations.

## Conclusions

Subpial hemorrhage occurs in neonates (especially term infants) in a variety of clinical scenarios. It has a unique MR image. For the first time, we identified three different patterns of MRI findings. All cases resembled the yin-yang sign or sandwich sign. The typical imaging pattern we described can be used to diagnose subpial hemorrhages or at least to suspect them. Subpial hemorrhage with underlying parenchymal hemorrhage suggests a greater relationship between venous compression and the pathogenesis of subpial hemorrhage.

Patients with Pattern A have good MRI prognoses, while patients with Pattern C develop subpial cystic cavities and encephalomalacia. Most patients have normal clinical outcomes. Further research is needed to gain a better understanding of the pathophysiology of this condition, define the range of risk factors, and determine the optimal treatment and expected long-term neurological outcomes.

## Data Availability

The datasets used and analyzed during the current study available from the corresponding author on reasonable request.

## Abbreviations

APTT: Activated partial thromboplastin time
AT3: Antithrombin
DWI: Diffusion-weighted imaging
D-D: D-Dimer
2D-TOF MRV: 2D phase non-contrast MR venography
FDP: Fibrin degradation products
FIB: Fibrinogen
FLAIR: Fluid-attenuated inversion recovery
INR: International normalized ratio
MRI: Magnetic resonance imaging
PACS: Clinical picture archiving and communication system
PT: Prothrombin time
SWI: Susceptibility-weighted imaging
TOF-MRA: Time-of-flight MR angiography
TT: Thrombin time
